# Advances in Ophthalmic Engineering—Integrating Biomechanics, Tissue Engineering, and Imaging for the Future of Vision Science

**DOI:** 10.3390/bioengineering12040374

**Published:** 2025-04-02

**Authors:** Sanfeng Xin, Zhuxin Xiong, Xiaofei Wang

**Affiliations:** Key Laboratory for Biomechanics and Mechanobiology of Ministry of Education, Beijing Advanced Innovation Center for Biomedical Engineering, School of Biological Science and Medical Engineering, Beihang University, Beijing 100191, China; ghostwolf@buaa.edu.cn (S.X.); xiongzhuxin@hotmail.com (Z.X.)

## 1. Introduction

Vision is one of the most essential senses, enabling individuals to interpret the world around them. However, ocular diseases and impairments continue to pose significant challenges globally. To address these challenges, the confluence of engineering and ophthalmology is crucial. This Special Issue exemplifies how cutting-edge engineering tools can help overcome the limitations of conventional ophthalmic diagnostics and therapeutics.

**Figure 1:**
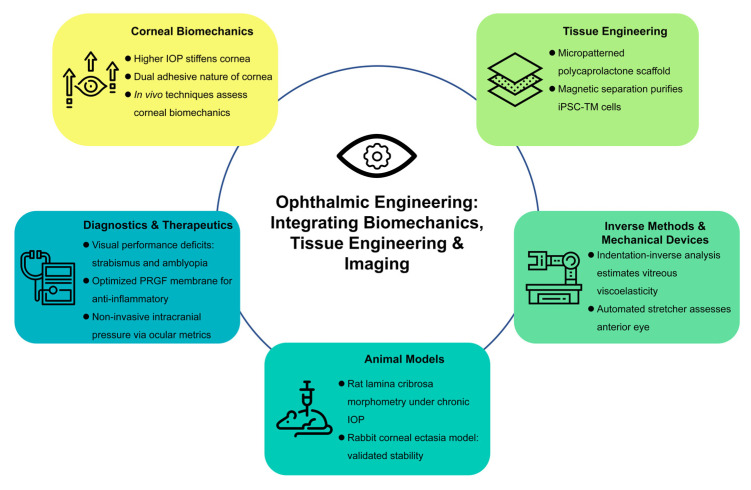
Integration of biomechanics, tissue engineering, and imaging in ophthalmic engineering.

In the following sections, we highlight the major contributions of the 12 articles included in this issue. The papers cover a wide spectrum of topics such as corneal biomechanics, trabecular meshwork engineering, inverse methods for determining mechanical properties, experimental animal models of ocular disorders, and novel diagnostic devices. By bringing together these varied yet complementary studies, this Special Issue not only underscores the multidisciplinary nature of ophthalmic research but also provides a comprehensive perspective on the future potential of engineering-driven innovations in vision science (Figure 1).

## 2. Advances in Corneal Biomechanics

Understanding the biomechanical behavior of the cornea is critical for diagnosing and managing diseases such as keratoconus. In the article by Ma et al. [1], the authors introduce a simplified artificial eye model to study the propagation of corneal surface waves under different intraocular pressures (IOPs). Using optical coherence tomography-based elastography, the study investigates how changes in IOP affect mechanical wave propagation speed and consequently the estimation of Young’s modulus. The authors demonstrated that an increase in IOP from 10 to 40 mmHg significantly increased wave propagation speeds and Young’s modulus. This work provides valuable insights into the relationship between IOP and estimated corneal stiffness and lays the groundwork for future technological development.

In vivo techniques to characterize corneal biomechanics are vital for diagnosing and managing conditions. The relationship between corneal morphology and biomechanics remains unclear, especially in diseased states, despite some established links. Various in vivo techniques, including air-puff tonometry, ultrasound biomicroscopy, optical coherence tomography, and finite element analysis, have been explored to assess corneal biomechanics, but no gold-standard measurement exists. Li et al. [2] reviewed these methods, their advantages and limitations, and their role in improving clinical decision making.

Moreover, Yang et al. [3] explored the intrinsic adhesive properties of the cornea, revealing that corneal adhesion exhibits dual characteristics akin to both solid and membrane structures. This discovery is particularly important when considering surgical interventions and the development of novel adhesives for corneal repair. The dual nature of corneal adhesion calls for more sophisticated models to simulate the biomechanical environment during wound healing and post-surgery recovery.

## 3. Tissue Engineering for Ocular Applications

The trabecular meshwork plays a key role in regulating aqueous humor outflow and maintaining normal IOP. Glaucoma, a major cause of irreversible blindness, is often associated with trabecular dysfunction. Beardslee et al. [4] made significant strides in this area by engineering a tissue that recreates the trabecular outflow pathway. Their study employed implantable, micropatterned, ultrathin, and porous polycaprolactone scaffolds to support the growth and function of trabecular meshwork cells. The study demonstrates that these scaffolds can be implanted successfully, promoting tissue integration and maintaining structural integrity, thereby offering a promising approach for trabecular meshwork regeneration. This approach represents a promising step towards cell-based therapies for glaucoma management, where the replacement of dysfunctional trabecular tissue may restore normal outflow and reduce elevated IOP.

Complementing these tissue engineering efforts, Feng et al. [5] reported the application of a magnetic platform in the purification of α6 integrin-positive induced pluripotent stem cell-derived trabecular meshwork (iPSC-TM) cells. The utilization of magnetic separation techniques enables the high-purity isolation of cells with a specific phenotype, which is crucial for both research and potential therapeutic applications.

## 4. Novel Experimental Methods and Inverse Analysis in Ocular Biomechanics

The quantitative assessment of ocular tissues is paramount for designing diagnostic tools and therapeutic strategies. Zu et al. [6] introduced an inverse method to determine the mechanical parameters of porcine vitreous bodies. Through the employment of an indentation test combined with inverse analysis techniques, the study provides a novel framework with which to estimate the viscoelastic properties of the vitreous humor. Such methodologies are invaluable because the vitreous plays a crucial role in ocular structure and function, and its mechanical properties can influence the onset and progression of retinal pathologies.

Along similar lines, Wang et al. [7] designed an automatically controlled multi-axis stretching device aimed at performing mechanical evaluations of the anterior segment of the eye. This device enables precise and reproducible mechanical tests, facilitating the quantification of ocular tissue responses to stretching. The innovation in designing an automated, multi-axis system not only minimizes operator variability but also accelerates data acquisition, making it a powerful tool for both research and clinical assessment.

## 5. Innovations in Animal Models and In Vivo Measurement Techniques

Animal models serve as essential platforms to bridge the gap between in vitro studies and clinical applications. Zhang et al. [8] investigated the morphological changes in the glial lamina cribrosa of rats subjected to chronically high IOP. Their findings shed light on the structural alterations that occur in response to sustained ocular hypertension—a key factor in glaucoma progression. The study’s rigorous morphometric analyses underscore the importance of using animal models to understand how chronic stressors affect ocular tissues at the cellular and structural levels.

Wei et al. [9] developed an animal model that simulates corneal ectasia in rabbits. Their study validates the stability and reproducibility of the model, which is essential for testing therapeutic interventions and for a better understanding of corneal pathophysiology. The availability of a reliable animal model allows researchers to investigate the progression of corneal disorders and to evaluate the efficacy of new treatments under controlled conditions.

## 6. Expanding the Frontiers of Ocular Diagnostics and Therapeutics

Another major theme in this Special Issue is the exploration of diagnostic and therapeutic methods. Ibrahimi et al. [10] compared the visual performances of patients with strabismus and amblyopia against those of healthy controls. Through detailed psychophysical and performance-based evaluations, the research provides insights into the specific deficits associated with these conditions. Such comparative analyses are critical for developing targeted rehabilitation strategies and for refining clinical assessment protocols to better tailor interventions to patient-specific needs.

Anitua et al. [11] focused on optimizing a plasma-rich growth factor (PRGF) membrane for the treatment of inflammatory ocular diseases. In fine-tuning the preparation and application of the PRGF membrane, the study demonstrates enhanced bioactivity and potential anti-inflammatory effects. This research not only broadens the therapeutic toolkit for managing ocular inflammation but also underscores the importance of biomaterials in regenerative medicine.

Furthermore, Dong et al. [12] provide a comprehensive review of the non-invasive methods for assessing intracranial pressure through ocular measurements. Recognizing the intricate relationship between ocular and cranial physiology, the review critically evaluates a variety of techniques to estimate the intracranial pressure through ocular measurement. This synthesis of current methodologies highlights the promise of ocular biomarkers as surrogates for intracranial pressure and paves the way for further refinements in non-invasive diagnostic devices.

## 7. Conclusions

The collection of 12 articles discussed in this editorial reflects the broad scope of current research efforts—from fundamental investigations into corneal mechanics to innovative approaches in tissue engineering and non-invasive diagnostics. Each contribution plays an integral role in advancing our understanding of ocular function and pathology, and together they point to a future where engineering solutions transform the clinical management of eye diseases.

As researchers continue to push the boundaries of what is possible, the integration of engineering principles into ophthalmology will undoubtedly lead to more effective diagnostic tools, personalized therapeutic strategies, and, ultimately, improved patient outcomes. The works presented in this Special Issue not only underscore the transformative potential of interdisciplinary research but also serve as invitations to the scientific community to further explore and expand upon these pioneering concepts.

This Special Issue is a testament to the power of interdisciplinary collaboration in driving innovation. It is our hope that the insights gained from these studies will inspire future research endeavors and ultimately lead to breakthroughs that safeguard and enhance the gift of sight for millions around the world.
